# Is DNA methylation of tumour suppressor genes epigenetic?

**DOI:** 10.7554/eLife.02475

**Published:** 2014-03-12

**Authors:** Kevin Struhl

**Affiliations:** 1 **Kevin Struhl** is an *eLife* reviewing editor, and is in the Department of Biological Chemistry and Molecular Pharmacology, Harvard Medical School, Boston, United Stateskevin_struhl@hms.harvard.edu

**Keywords:** CpG island methylator phenotype, INK4-ARF, colorectal cancer, ZNF304, KRAS, DNMT1, human, mouse

## Abstract

In colorectal cancer cells, a non-epigenetic transcriptional pathway that is mediated by an oncogene maintains DNA methylation of tumour suppressor genes

**Related research article** Serra RW, Fang M, Park SM, Hutchinson L, Green MR. 2014. A KRAS-directed transcriptional silencing pathway that mediates the CpG island methylator phenotype. *eLife* **3**:e02313. doi: 10.7554/eLife.02313

**Image** The instructive model of DNA methylation, which inhibits the expression of tumour suppressor genes

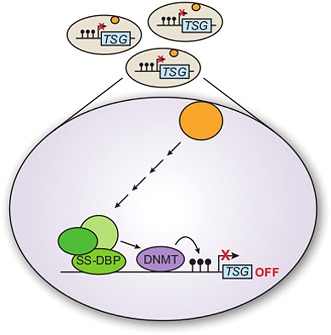

Many cancers are a result of genetic changes that either inactivate a tumour suppressor gene or create an oncogenic form of a normal gene. However, a suppressor gene can also be inactivated by a process called DNA methylation, which involves an enzyme called DNA methylase adding methyl groups to sites at or near the gene (Baylin and Jones, 2011). This process is said to be ‘epigenetic’ because the sequence of bases in the DNA is not changed.

However, the term epigenetic does not simply mean a non-genetic alteration that influences phenotype; it also encompasses the idea of inheritance. This consideration leads to two questions. First, how do tumour suppressor genes get methylated? Second, how is DNA methylation of tumour suppressor genes inherited through multiple generations? Now, in *eLife*, Michael Green and colleagues at the University of Massachusetts—including Ryan Serra and Minggang Fang as joint first authors—build on previous work performed in the Green laboratory (Gazin et al., 2007; Palakurthy et al., 2009; Wajapeyee et al., 2013) to demonstrate that DNA methylation of tumour suppressor genes is controlled by a transcriptional regulatory pathway that is triggered by an oncogene. These findings raise the question of whether DNA methylation really is epigenetic (Serra et al., 2014).

There are two basic models for how tumour suppressor genes can be methylated: the stochastic model and the instructive model (Figure 1). In the stochastic model, which is implicitly favored in the literature, methylation of tumour suppressor genes occurs by chance, with the resulting cells having a selective growth advantage. During the replication of DNA that has been methylated, each new molecule of DNA is hemi-methylated: that is, one strand is methylated and one strand is not. DNA methylases then add methyl groups to the strands that are not methylated, thereby maintaining the methylation pattern in the next generation (Figure 1A). One problem with the stochastic model, rarely if ever considered, is that the methylated sites on tumour suppressor genes are commonly located in close proximity, and it is unclear how this could occur by a random process.

**Figure 1.**
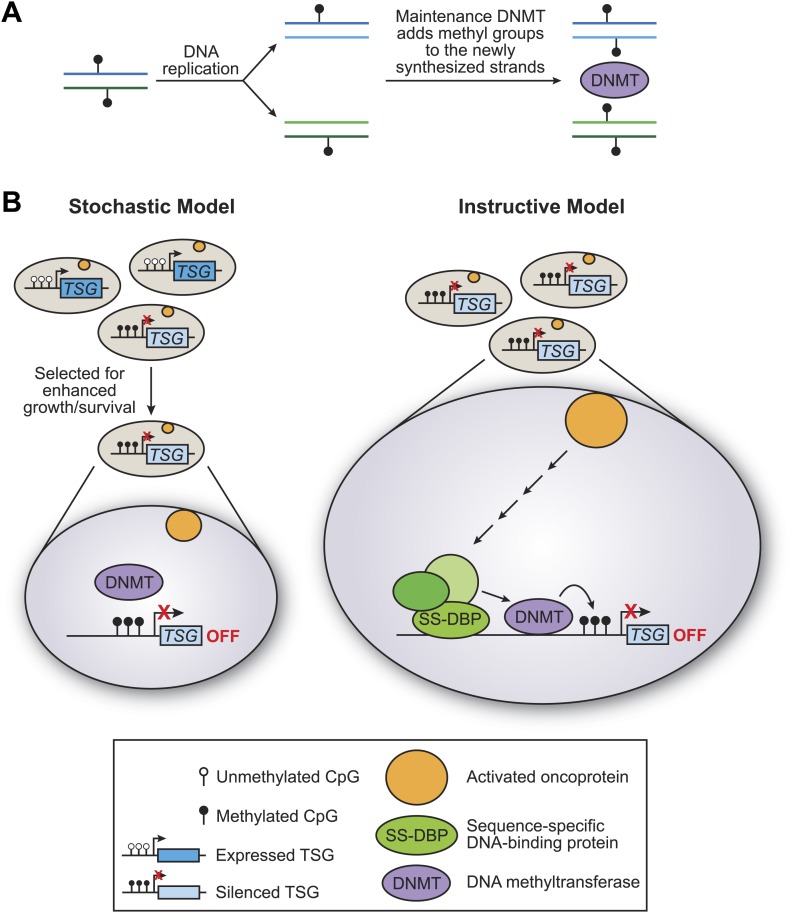
The stochastic and instructive models for DNA methylation of tumour suppressor genes. (**A**) Prior to replication, both strands of the DNA are methylated at CpG sites. Upon DNA replication, the parental strands remain methylated, but the newly synthesized strands are not. DNA methylases (DNMT) add methyl groups to these hemi-methylated CpG sites, thereby maintaining the methylation pattern at this location in the next generation. (**B**) In the stochastic model (left), a cell containing methylated CpG sites and a silenced tumour suppressor gene (TSG) occurs by chance and is selected for enhanced growth/survival. Methylation of CpG residues is maintained by methylases as described in (**A**). In the instructive model (right), an activated oncoprotein (yellow circle) directs DNA methylation of the tumour suppressor gene via a classical transcriptional pathway involving a sequence-specific DNA binding protein (SS-DBP), a co–repressor complex (green circles) and DNA methylase. The loss of any component in this pathway results in the loss of DNA methylation and the increased expression of the tumour suppressor gene. The results of Serra et al. support the instructive model: these experiments also show that the pathway starts with an activated KRAS enzyme (shown here as a yellow circle) stimulating the expression of a kinase called PRKD1 and its substrate USP28 deubiquitinase (not shown). Phosphorylated USP28 removes a ubiquitin group from a transcription factor called ZNF304, thus increasing its concentration in the nucleus. This transcription factor (shown here as SS-DBP) binds to a specific region of DNA and recruits a scaffold protein called KAP1, an enzyme called SETDB1 that methylates histones, and a DNA methylase called DNMT1.

In the instructive model, an oncogene starts a series of specific molecular events that culminates in DNA methylation of the tumour suppressor gene (Figure 1B). The key distinction between the two models is whether the DNA methylase is specifically targeted to the tumour suppressor genes or instead non-specifically methylates sites on a genome-wide basis. Such targeted or non-targeted modes of action are analogous to those of histone acetylases and other chromatin-modifying activities that are involved in transcriptional regulatory mechanisms (Struhl, 1998). In particular, classical transcriptional activation and repression mechanisms involve the targeted recruitment of chromatin-modifying activities to specific genes, whereupon they create local domains of histone modifications and either increase or decrease the transcriptional output.

The recent work by Green and colleagues utilised a genome-scale RNA interference screen to identify genes that are critical for silencing one particular gene location—the hypermethylated *INK4-ARF* tumour suppressor locus—in a human colorectal cancer cell line (Serra et al., 2014). This cell line, like some other cancer cells, contains a mutation of the gene encoding a protein called KRAS.

Detailed molecular analyses of these genes (and their gene products) in several colorectal cell lines with this mutation, and in diseased tissue samples, reveal the following pathway (Figure 1B). The KRAS protein stimulates enzymes that ultimately inhibit the degradation of a transcription factor called ZNF304, thereby increasing its concentration in the cell nucleus. This transcription factor can then recruit a co-repressor complex that includes a DNA methylase and two other proteins (Figure 1B). The end result is that the DNA is methylated at a particular location and that transcription of the tumour suppressor gene is repressed.

This pathway is a classical transcriptional mechanism where DNA-binding proteins recruit chromatin-modifying activities, but with the additional twist of the recruitment of the DNA methylase and the subsequent DNA methylation. Therefore, maintenance of DNA methylation requires the continued presence of the proteins that are directly associated with the target locus, or are required for this association. The inactivation of any of these proteins leads to the loss of DNA methylation and transcriptional silencing. Lastly, and again typical of transcriptional mechanisms, the pathway affects not just *INK4-ARF*, but plays a key role in the methylation of around 50 other genes.

This and an earlier example in a mouse cancer cell line (Gazin et al., 2007; Wajapeyee et al., 2013) strongly suggest that, like histone modifications, DNA methylation is not epigenetically inherited. Instead, it is maintained by an instructive transcriptional mechanism that represses multiple genes. As methylation is greatly diminished if any component of the transcriptional pathway is inactivated, non-recruited DNA methylases (sometimes called maintenance DNA methylases) are not sufficient to propagate methylated tumour suppressor genes.

More generally, epigenetic inheritance is determined primarily by transcriptional circuitry, and not by histone modifications or DNA methylation (Ptashne, 2013). For example, induced pluripotent stem cells (Takahashi and Yamanaka, 2006; Young, 2011) and muscle cells (Weintraub, 1993) can be generated from other cell lineages by introducing the critical transcription factors that trigger positive feedback loops involving the endogenous factors that define the relevant cell type. In fact, the reproductive states of bacteriophage λ, a virus that infects *E. coli*, are epigenetically maintained in the absence of chromatin (Ptashne, 2013).

The strong preference of DNA methylases for hemi-methylated substrates undoubtedly contributes to the stable maintenance of hypermethylated tumour suppressor genes (such as INK4-ARF). By analogy, other chromatin-modifying repressors both introduce repressive histone modifications and preferentially recognise chromatin harbouring the modifications they introduced. These include the yeast Sir proteins, which remove acetyl groups from histones; HP1, which methylates the H3-K9 site; and the polycomb complexes, which methylate the H3-K27 site. Thus, both DNA methylation and histone modifications are generated by targeted recruitment of enzymatic activities, and these modifications serve a reinforcing, but not instructive, role in maintaining epigenetic states. While they are clearly not sufficient, non-recruited DNA methylases could also contribute to maintaining the hypermethylated repressed state by providing an independent methylation mechanism that depends on the previous methylation status.

Serra et al. and the previous studies have elucidated the first molecular mechanism for the methylation of tumour suppressor genes, namely maintenance by an instructive transcriptional mechanism that represses multiple genes. The inability of non-recruited DNA methylases to maintain DNA methylation in cancer cells when this transcriptional mechanism has been inactivated casts doubt on the stochastic model and on DNA methylation being epigenetically inherited. Although these are only initial examples, the general finding that DNA methylation occurs at multiple sites over a localised region strongly argues that this instructive model of DNA methylation is a widespread, and perhaps universal, principle in cancer biology.
